# Dual practice in the health sector: review of the evidence

**DOI:** 10.1186/1478-4491-2-14

**Published:** 2004-10-27

**Authors:** Paulo Ferrinho, Wim Van Lerberghe, Inês Fronteira, Fátima Hipólito, André Biscaia

**Affiliations:** 1 Associação para o Desenvolvimento e Cooperação Garcia de Orta, Lisbon, Portugal; 2 World Health Organization, Geneva, Switzerland

## Abstract

This paper reports on income generation practices among civil servants in the health sector, with a particular emphasis on dual practice. It first approaches the subject of public–private overlap. Thereafter it focuses on coping strategies in general and then on dual practice in particular.

To compensate for unrealistically low salaries, health workers rely on individual coping strategies. Many clinicians combine salaried, public-sector clinical work with a fee-for-service private clientele. This dual practice is often a means by which health workers try to meet their survival needs, reflecting the inability of health ministries to ensure adequate salaries and working conditions.

Dual practice may be considered present in most countries, if not all. Nevertheless, there is surprisingly little hard evidence about the extent to which health workers resort to dual practice, about the balance of economic and other motives for doing so, or about the consequences for the proper use of the scarce public resources dedicated to health.

In this paper dual practice is approached from six different perspectives: (1) conceptual, regarding what is meant by dual practice; (2) descriptive, trying to develop a typology of dual practices; (3) quantitative, trying to determine its prevalence; (4) impact on personal income, the health care system and health status; (5) qualitative, looking at the reasons why practitioners so frequently remain in public practice while also working in the private sector and at contextual, personal life, institutional and professional factors that make it easier or more difficult to have dual practices; and (6) possible interventions to deal with dual practice.

## Introduction

Doctors and nurses in government employment are labelled "unproductive", "poorly motivated", "inefficient", "client-unfriendly", "absent" or even "corrupt". These labels are often associated with coping strategies associated with widespread "demotivation", due partly to "unfair public salaries". These are presented as the de facto justification of "inevitable" predatory behaviour and public-to-private brain drain [1-6]. In many countries, developed and developing alike, this has eroded the implicit psychological and social contracts that underlie the civil service values of well-functioning public organizations [7]. As a result, public servants often resort to dual or multiple employment.

This paper reports on income generation practices among civil servants in the health sector, with a particular emphasis on dual practice. It first approaches the subject of public–private overlap. Thereafter it focuses on coping strategies in general and on dual practice in particular.

### Public–private overlap

Private providers capture a significant and growing share of the service delivery market for health care, and ensure an important part of the uptake of services. For a sample of 40 developing countries, an average of 55% of physicians worked in the private sector and an average of 28% of health care beds were private beds (21% private, for profit) [8].

Asia has more than 60% of private sector contributions to health care financing (excluding China and India) and is the part of the world where the private sector normally plays the dominant role. In Malaysia, for example, the proportion of physicians in private practice increased from 43% in 1975 to 70% in 1990. In Indonesia half of the hospitals are privately run. In Thailand the share of beds in private hospitals grew from 5% in 1970 to 14% in 1989.

The contribution of the private sector to health care financing in Africa is 50%, with a significant preponderance of private, not-for-profit, nongovernmental (PNFP-NGO) entities, particularly church organizations. In Zimbabwe church missions provide nearly 70% of all beds in rural hospitals and in Tanzania they run 40% of the hospitals. In Kenya, about one third of the total health services and 40%–50% of the family planning services are provided by PNFP-NGOs. In Latin America the private sector finances 40% to 60% of the health sector [9].

For many populations, especially in rural areas, these PNFP-NGOs are the main – if not the only – providers of health care. For example, in a mail survey of 88 nongovernmental hospitals in sub-Saharan Africa at the end of the 1980s, 39 were the only service provider in their district [10,11]. It is also the case, for example, of rural Congo in the 1990s, where only NGO-supported districts continued working [12]. Following the reforms of the early 1990s in Mali, most ambulatory health care – nearly all in rural areas – is now provided by community-owned PNFP health centres organized in a nongovernmental federation [13]. But these are mainly rural situations.

In urban areas PNFP-NGOs usually share the work with private, for-profit (PFP) providers and government services. In Alexandra, South Africa – a poor periurban township – for example, a PNFP university clinic, a municipal clinic and PFP "general dispensing cash practices" worked side by side [14,15]. But in general, the market share of private providers is more limited.

PFP health providers are no doubt an important source of ambulatory care throughout the developing world, but tend to concentrate on the more profitable niches of the market. The development of private practice in most developing countries is notoriously unregulated. Private practices are not easily forthcoming with information, at times for fear of tax implications, at times because existing regulations are not respected, often because of a lack of respect for discredited ministries of public health and not infrequently because of the non-existence of information systems. In transitional countries, such as Tunisia, the growth of the entrepreneurial sector is well documented.

Although hard data are hard to come by for the poorer countries, it is sufficient to walk around Luanda or Dar es Salaam or to look at the advertising sections of any newspaper in Maputo to see that private health care provision is a thriving growth industry. If there are ever more private providers on the market, not all provide the whole range of health services. They tend to select niches according to demand and competition rather than providing comprehensive care.

There are huge differences between and within countries. This (patchy and scarce) evidence confirms that both PFP and PNFP providers have a significant and growing share of the market of health care, but this statement needs qualification. There are wide differences between and within countries. Overall, the market share of the private sector is smaller for inpatient than for ambulatory care, and limited for preventive and public health services [8].

### Coping strategies

Individual coping strategies represent the health professionals' ways of dealing with unsatisfactory living and working conditions. In many countries their prevalence has increased over recent years. The notion of the full-time civil servant exclusively dedicated to his/her public sector job is disappearing. Were this without consequences for the performance of the public health sector, it would not be much of a problem. Not all coping strategies can be classified as predatory behaviour or corruption, and their effects on the health care system can be positive as well as negative. They cannot, however, be ignored as, in many countries – particularly the poorest – the situation has gotten out of hand.

Most would agree that public sector salaries are most often "unfair". For example, in 1999 a Mozambican nurse's salary was only 10% to 15% of what it had been 15 years before [16]. In Sierra Leone most health professionals, physicians included, are employed by the public sector at salaries under USD 100 per month [17]. In many other countries health staff is going through similar experiences. In Russia, state doctors earn between USD 15 and USD 50 per month [18]. In the Dominican Republic, in 1996, physicians with 20 years of experience earned the same as new medical graduates, rewards for good performance were impossible and personnel were paid regardless of whether they performed their duties [19]. In such a context, demotivation, overall lack of commitment and low productivity are to be expected.

To compensate for unrealistically low salaries, health workers rely on individual coping strategies [1-6,18,20-54]. Many clinicians combine salaried public sector clinical work with a fee-for-service private clientele [3-6,25-29,32,38-41,44,45,53]. Others resort to absenteeism [24,44,45], or predatory behaviour, asking under-the-counter payments for access to services intended to be free of charge [33,34,46] or goods and/or misappropriating drugs or other supplies [20,21,27,43,49] and referral of public sector patients to private practices [23].

In Thailand 37% of patients pay their public sector obstetricians "gratitude money" [55]. In France, mainly for surgical interventions, under-the-table remuneration is very common in hospital or ambulatory settings [56]. In Greece, doctors and nurses have been criticized for receiving gifts and bribes [57].

Another example is fee splitting, whereby a specialist shares a fee with the referring physician [23]. In 1998, for example, a group of Italian general practitioners was suspended for accepting payments to send their patients to a particular private centre for radiology examinations [58]. It is common practice in the United Kingdom for consultants to spend time in private clinics when they should be attending to their public duties [23].

Patients and practitioners may collude to deceive a third agent: in Kazakhstan, for example, it is reported that doctors regularly provide false health reports in return for a fee, so that patients can obtain driving licenses [23]. A further form of fraud through misinformation is exemplified by the case of the English GP who forged consent forms for patient participation in medical trials in order to boost income [53]. The problems these coping strategies create are increasingly recognized [6,38-40]., although the subject remains taboo for many ministries of health and development agencies.

Drugs are – in the current context of scarce resources, health care reform, promotion of generics, the HIV epidemic and growing demand for health care – a sensitive issue, as in many low-income countries, pharmaceuticals make up 50% or more of health care costs [59]. With health sector reforms, private sector pharmacies are increasingly becoming the first and sometimes the only outlet for the delivery of health services [60-63]. In this environment, and for several reasons – including the "business profession dilemma" in private pharmacy practice – irrational prescription can become a major problem [64-67]. Antibiotics are often sold without a prescription [68,69].

In other settings, such as hospitals and health centres, misappropriation is a widespread practice by all categories of professionals. This is infrequently acknowledged explicitly or documented, even in studies that have looked into the coping strategies of health professionals [3-5,28,29,41,44,45]. It has nevertheless been documented in a number of African [20,21,27,43,49] and Latin American countries [22]. Where documented it is perceived as common practice.

In Uganda, for example, misuse of pharmaceuticals was reported by facility health workers as well as by the District Health Teams and the Health Unit Management Committees; resale of drugs represented the greatest single source of income for health workers in most units [27,43]. In many other developing countries the situation is supposed to be similar if not worse.

## Dual practice

We have stated that many clinicians combine salaried public-sector clinical work with a fee-for-service private clientele [3-6,25-29,32,38-41,44,45,53]. Dual practice may be considered present in most countries, if not all. Nevertheless, there is surprisingly little hard evidence about the extent to which health care workers resort to dual practice, about the balance of economic and other motives for doing so, or about the consequences for the proper use of the scarce public resources dedicated to health. Dual practice is often a means by which health workers try to meet their survival needs, reflecting the inability of health ministries to ensure adequate salaries and working conditions.

In this paper dual practice is approached from six different perspectives: (1) conceptual, regarding what is meant by dual practice; (2) descriptive, trying to develop a typology of dual practices; (3) quantitative, trying to determine its prevalence; (4) impact on personal income, the health care system and health status; (5) qualitative, looking at reasons for its prevalence and for staying in public practice while working in the private sector and at contextual, personal life, institutional and professional factors that make it easier or more difficult to have dual practices; and (6) possible interventions to deal with dual practice.

### What is meant by dual practice

Dual practice is approached in the literature with great diversity. It can mean health professionals with multiple specializations – for example, among Egyptian physicians the most popular areas of multiple specializations are "cardiology and internal medicine, internal medicine and fever and cardiology and chest" [70].

It can also mean health professionals working within different paradigms of health (allopathic medicine combined with traditional medicine – Chinese, African or otherwise – or with other paradigms such as osteopathy, homeopathy and others). It may involve combining different forms of health-related practice – clinical with research, with teaching or with management. It can also mean health professionals combining their professional health practice with an economic activity not related to health (such as agriculture).

In this paper, dual practice will be used to describe multiple health-related practices in the same or different sites.

### An operational typology of dual practices

Therefore, in terms of sector location, dual practice may be public on public, public on private or private on private (Table 1).

**Table 1:** Typologies of dual practice

	**Public**	**Private, for-profit**	**Private, not-for-profit**
Public	+	+	+
Private, for-profit		+	+
Private, not-for-profit			+

Overtime may be considered a form of dual employment. It is increasing because of cost-containment measures or shortages of staff. In 1992, the German Union of Salaried Employees estimated that the overtime worked by health workers was equivalent to 20 000 extra full-time staff posts. Today, trade unions in Canada and the United Kingdom express particular concern about the use of overtime to substitute for recruitment and about the increase in unpaid overtime [71].

### The extent of dual practice

As mentioned and supported by the limited literature available (Table 2), dual practice is probably present in all countries regardless of income, even in settings – such as China – where there are major regulatory restrictions [29,55,70,72-82]. In Latin America physicians usually hold jobs in both public/social security and private systems [83].

**Table 2:** The extent of dual practice in several countries

**Country**	**Type and frequency of dual practice**
Angola	Dual (public and private) practice is ubiquitous and unregulated [72].
Cambodia	Dual (public and private) practice is ubiquitous [89].
Egypt	Rural-based Egyptian physicians in private practice are more likely to have a second job (85%) than urban-based physicians (71%). However, there is not much difference between urban and rural physicians in the likelihood of having a third or fourth job: 15% of urban and 11% of rural physicians have a third job and 2% of urban and 1% of rural physicians have a fourth job. Twenty percent of 113 single private practice dentists work only in their private clinic; 73% have 2 jobs, 6% have 3 jobs and 1% have 4 jobs. Among 261 pharmacists 91% have only one job, 8% have two jobs and 1% have 3 jobs. Among 80 other health service providers, mainly unlicensed, who are officially not allowed to operate, but yet provide a significant amount of health care, 66% of the sample have a second job and 1% has a third job [70].
Indonesia	Most doctors have dual practices in the public and private sectors [77].
Malawi	The government allows serving medical personnel in its facilities to set up private surgeries where they can practice after official duty hours; it further allows those without professional qualifications (e.g. "paramedics") to set up a health care business for minor health complaints [78].
Mozambique	Common among urban, but not rural, health professionals [29].
Papua New Guinea	Semi-private wards in public facilities are well patronized in the larger hospitals but tend to be underutilised in the smaller provincial centres [79].
Peru	Almost all physicians have both public and private practices [77].
Portugal	23% of public sector health centre workers have a second job, the highest rate being for doctors – 43%; 58% of public sector hospital workers have a second job, the highest rate being for doctors – 50% [75, 76,75, 76].
South Africa	Half of general practitioners in private practice have other employment. While 36% worked in the public sector, this was more common in rural (62%) compared with urban (21%) areas [80].
Syria	Most physicians have dual practice [77].
Thailand	An estimate suggested that in Bangkok alone there were over 2000 private clinics, many of these run by government doctors [81]. Private practice by public sector obstetricians is very frequent [55].
Viet Nam	Most doctors complement public sector work with private practice [77]. Full-time government employees are supplementing their incomes through part-time private practice. One village-based health survey found that 70% of the drug sellers were moonlighting government workers [82].

### The impact of dual practice

#### Predatory behaviour

Dual practice may lead to predatory behaviour (behaviour in which self-gain is pursued to the detriment of the legitimate interests of colleagues, services and/or patients)[84] by health workers. This is particularly strong in situations where market conditions – usually high physician supply, as is the case of capital cities in Africa and other urban more than rural areas – would otherwise reduce their incomes. In these situations clinicians use their authority to prescribe treatment for their patients to generate additional demand for their own services.

This hypothesis, controversial for some practices such as caesarean sections, seems consensual regarding other surgical practices [85]. In Thailand, for example, it is well demonstrated that antenatal care is sought in private ambulatory facilities, while caesarean sections are offered in public hospital facilities. Caesarean-section rates among private patients (46%) are three times higher than among non-private patients (16%), which indicates that private practice by public obstetricians is a strong determinant of caesarean sections [55].

The predatory behaviour of individual clinicians constitutes, in many cases, a de facto financial barrier to access to health care [21]. More important, in the long run, is that it delegitimizes public sector health service delivery and jeopardizes the necessary relation of trust between user and provider [20].

#### Conflicts of interest

A more insidious problem is that of conflicts of interest. Effects on the system can best be looked at separately for each type of side activity. When health officials set up in dual practice to improve their living conditions – or merely to make ends meet – this may not interfere with their work as civil servants (although it is likely to compete for time and to reinforce rural-to-urban migration). When they take up teaching as an extra job, usually in public sector institutions, that may actually be beneficial to the public agenda, as it reinforces the contact of trainees with the realities of the health services.

For doctors who are basically managers, moonlighting in private practice presents less of a conflict of interest than for clinicians. The latter must compete for patients with themselves, and thus they have an incentive (and the opportunities) to lower the quality of the care they provide in the public services. This is not the case for managers: involvement in NGO projects or work for donors can foster better coordination in the provision of services, but may constitute a conflict of interest when NGO or project policies are not necessarily congruent with national health policies or the agenda of the public service [4,44,52,55].

Other business activities, such as agriculture, are neutral towards health services, although they may constitute a de facto internal brain drain [3].

#### Brain drain

Dual practice is to a large extent a question of creating and making use of opportunities. The pursuit of such opportunities contributes also to brain drain.

Brain drain of health professionals is often thought of only in terms of intercountry migration [86]. But failure to post and retain the right person at the right place is not merely a question of a Congolese doctor's deciding to move to South Africa or a Philippine nurse's moving to the United States. It is also a question of internal – including public to private sector – "migration".

This public-to-private migration compounds the rural-to-urban migration because cities also offer more opportunities to diversify income generation [28,29]. The need to make up for inadequate salaries – and to be in a setting where there are opportunities to do so – thus fuels rural-to-urban migration and resistance against redeployment to rural areas [4,28,29]. Professionals who have successfully taken advantage of these urban opportunities increase their market value over time, until they are ready to leave public service. Rural-to-urban brain drain thus is later compounded by public-to-private brain drain.

#### Competition for time and limits to access

Coping strategies, including dual practice, also affect access, but through competition for time. In many countries, civil service medical staff is available only nominally full-time to fulfil its assigned tasks. This has been well described regarding Colombia, Costa Rica and Venezuela. In Venezuela doctors and head nurses missed about one third of their contracted service hours, 37% and 30% respectively, while resident doctors and nurses were absent about 7% and 13% of the time, respectively [47]. In Peru 32% of doctors and nurses considered absenteeism common or very common [24]. In Costa Rica 65% of doctors and 87% of nurses felt that physicians were unjustifiably absent from work or, even if present at work, often saw private patients on public time in public facilities [22]. Absenteeism in the hospitals of Bogotá, Colombia, may cost over USD 1 million a year [35].

If public sector medical staff is moonlighting in private practice, this evidently limits access by public patients. This corresponds to a net flow of resources out of the public sector. Competition for time is also something that concerns managers, whose coping strategies are often more oriented towards collaboration with development agencies [44,45].

Competition for time is a nagging problem for many development agencies and ministries of health. At times it is blatant. In Mali, for example, regional health staff was found to spend 34% of its total working time in (income-generating) workshops and supervision missions supported by international agencies; for chief medical officers it was 48% (El Abassi & Van Lerberghe, unpublished data, 1995). The 73% of working time spent on official duties that was self-reported by the respondents to one of the surveys reviewed may well be an overly optimistic estimate [44,45].

In Egypt the number of hours worked in the private clinic falls as the number of a physician's jobs increases, indicating that the physician replaces hours away from the private clinic with other employment. Secondly, as the number of jobs increases, the amount of time spent in the second job, usually a government job, decreases. This reduces the access of low-income people to medical care, as they cannot afford to seek care in the private sector.

Econometric analysis of the data also found that physicians replace hours they should be working at the government job by hours in the private clinic. This has important policy implications, as multiple employment is not increasing the access of the population to medical care. On average, physicians see one patient per hour in their private clinic. This increases to 3.2 patients per hour in the second job, 4.4 in the third job and 2.9 in the fourth job. An extreme example is that of general practitioners who see one patient per hour in their private clinic, approximately four in the second job and 12 in their third job. In general, the number of patients seen per hour increases with the job number. This is true for physicians as well as dentists [70].

Competition for time automatically results in a transfer of salary resources out of the public sector through reduced availability – at least the equivalent of 27% of the salary mass [44,45].

#### Outflow of resources

Besides competition for time, in many cases the use of the public sector's means of transportation, office infrastructure and personnel represent additional hidden outflows of resources, often in association with dual practice. The overall impact of this outflow of resources is hard to quantify in any country. Reports from Moscow suggest that up to 30% of the federal budget is not accounted for [18,34]; in the United Kingdom, estimates of GBP 115 million are given for prescription fraud alone [23].

The loss to the public sector associated with redirection of diagnostic and therapeutic resources, such as pharmaceuticals, to private practice or into the black market is obviously difficult to assess. In Uganda, for example, it results in a significant loss to the public health facilities: the median drug leakage in health facilities was estimated at 78% [27,43]. In the Dominican Republic, almost one third of total hospital expenditure remains unaccounted for, representing some combination of theft of materials and supplies, diversion of funds and gross mismanagement [37]. In Panama, high-value medications were stolen on a daily basis, with significant losses to the hospital [87]. In Venezuela, between 10% and 13% of all medical supplies and medications were stolen [47].

In Costa Rica, 71% of doctors and 83% of the nurses reported that equipment and materials had been stolen in their hospital [22]. In the United Kingdom it is estimated that pilfering – of bandages, medications and stationery, for example – adds up to more than GBP 15 million annually. In an Andalusian hospital in Spain, it was estimated that pilfering of food supplies led to per capita catering costs that were higher than those of a good restaurant [23]. The ultimate purpose of this stealing is not studied, but it is possible to speculate that many stolen resources find their way into the dual practices of public servants.

Competition for time and transfer of resources are compounded by the fact that the best-trained and most competent officials are also the most likely to divert their time to other activities outside the health sector (a de facto brain drain). This in turn reinforces the attraction of what starts out as a job-on-the-side, and quickly becomes not only more rewarding financially but also professionally and in terms of social prestige.

The impact of these coping strategies is seen as negative. These strategies weaken the public sector health structure and damage people's health [20,88].

#### Impact on income

**Figure 1:**
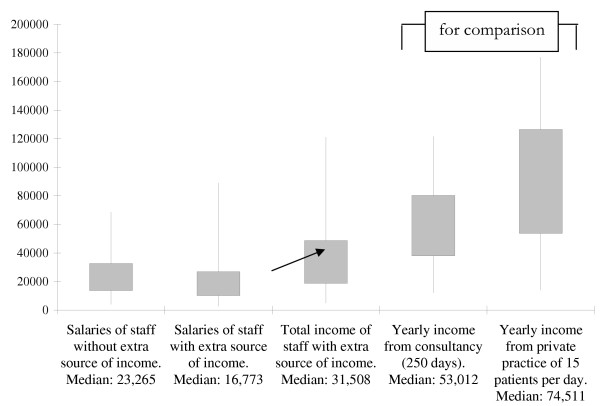
Distribution of income in USD purchasing power parity, with the increase from extra jobs, compared to distribution of potential income through consultancies or private practice

Individual income topping-up strategies allow professionals a standard of living that is closer to what they expect. In one study, these strategies more than doubled the median income of public sector health managers, and brought it from 20% to 42% of that of a full-time private practice [44,45] (Tables 3 and 4. Fig. 1: The box-plot chart represents for each variable the maximum, 75th percentile, 25th percentile and the minimum [44,45].)

**Table 3:** Median and interquartile range of take-home salaries of civil servant health service managers

	**Low-income countries (61 respondents)**	**Middle-income countries (39 respondents)**
In USD at official exchange rate	3802 (2137–5249)	11 253 (6704–18 900)
In USD corrected for purchasing power parity	13 890 (9411–20 956)	26 376 (18 416–38 931)
As % of the income of a private practice serving 15 patients per day	14% (10%–33%)	29% (22%–41%)
As % of the income of full-time consultancy work (250 days / year)	31% (23%–44%)	81% (45%–108%)

Source: [44, 45]

**Table 4:** Median and interquartile range of total income (salary plus extra activities) of civil servant health service managers

	**Low-income countries (61 respondents)**	**Middle-income countries (39 respondents)**
In USD at official exchange rate	5899 (2712–8137)	11 372 (6000–23 040)
In USD corrected for purchasing power parity	21 438 (4081–84 640)	39 377 (26 149–64 338)
As % of the income of a private practice serving 15 patients a day	26% (17%–52%)	42% (29%–64%)
As % of the income of full-time consultancy work (250 days/year)	49% (30%–96%)	115% (74%–172%)

Source: [44, 45]

In Phnom Penh, Cambodia, 90% of a physician's income in dual practice is derived from the private sector activity [89]. In Thailand earnings from private practice among physicians constitute 55% of their total income [55]. The upside is that income topping-up helps to retain valuable expertise in public service [90-96].

#### Corruption in the health sector

Corruption has been a long-standing concern in development circles. The literature is rich on theories ranging from macrosociological analyses of sociocultural processes to dyadic game theory modelling. Although impressive, this theorizing has not resulted in useful, empirically validated tools to redress this problem [97].

For these reasons, we prefer to understand this issue from the perspective of the context that expects health professionals to have a standard of living that cannot be met by existing social systems, sometimes to a level where they cannot even satisfy their basic needs through their public sector salary.

In Mozambique, for example, medical students seem to know they will be needed in the public sector, and that this would represent an opportunity to contribute to the public's welfare. Nevertheless, in order to improve their earnings their expectations are to combine their public sector practice with private medical work. One third of the medical students expect an income of between USD 715 and USD 1071 a month, and another third expects over USD 1429, at a time when the salary of a newly graduated doctor is about USD 357 a month. This sets the scene for the reality, often unregulated, of dual practice that plagues many countries [98]. It is not surprising that once graduated they resort to dual practice (often already initiated as medical students).

One must distinguish between individual coping strategies and orchestrated activities, acknowledging, nevertheless, that they may be closely interrelated. For example, hospitals with limited budgets in Mozambique, Portugal, Russia and other countries see dual practice as a means of retaining the most senior personnel.

There is a fine line separating coping strategies, including dual practice, from corruption. The difficulty in differentiating between the two starts with the definition of corruption. Widely accepted definitions often have ideological connotations. The definition of corruption as the "private use of public goods" is frequently associated with authors who ultimately defend a greater role for the private sector in the provision of public services such as education and health (as proposed by Van der Geest) [21], but without sufficient evidence of its effectiveness. They fail to acknowledge that corruption in the private sector may be a significant problem [99] and that liberalization and transition from state-controlled systems to systems in which the market plays a greater role have often resulted in more corruption, not less [100].

Another problem with current research is its treatment of corruption as something that would be the same everywhere, with essentially the same causes and implications wherever it occurs [101].

A third problem is that of the moralistic and criminal connotations of the word "corruption". It should be kept in mind that not all that is illegal is corrupt and not all that is corrupt is illegal, and that also a distinction should be maintained between corrupt transactions and those that are immoral [99].

The literature tends to focus on the "corrupt", failing to acknowledge that contexts that generate so-called corrupt behaviours generate them across the whole spectrum of society, to the extent that they become an ingrained and acceptable part of society, a necessary evil to survive in a very harsh environment. This shift from focusing on the persons involved to the system in which the professionals are integrated has taken place in other areas, such as in quality management [102] and in the prevention and control of industrial accidents [103] or medical errors [104]. Sooner or later this is likely to happen with the corruption literature as well.

Corruption has a negative impact on development in general. It hits the poor the hardest, directly and indirectly by, inter alia, reducing their access to public services such as education and health care [105,106].

This negative impact is also felt in indicators of health status. There is an inverse relationship between indices of corruption and ratio of public health spending to GDP and child mortality rates [107]. As such, it cannot be ignored by health sector managers, but labelling is not only misleading and counterproductive: it also does not help mobilize the coalitions necessary to address the problem.

When compared with other sectors, health is frequently classified as being of median to high levels of corruption, with sectors such as public works contracts and construction, arms and defence, energy and industry appearing as more corrupt [108].

The professional literature hardly touches on corruption in the health sector. It is anecdotal (Yudkin reports, from two Kenyan newspaper articles, that two ministry of health officials had been bribed to purchase sufficient quantities of two medicines made by one company to last the nation for more than 10 years, whereas at least one of the medicines would expire in two to three years) [109] (see also Baxter, 1998) [110], biased, peripheral to the core issues [22] or lacking in empirical data. Empirical data are, nevertheless, available from a number of studies and reports [16,18,21-23,105,110-113], most of which were reviewed in previous sections.

One of the earlier papers on this was by Van Der Geest [21]. It attempted to explain why health services in southern Cameroon functioned so inefficiently, with special attention to the distribution of medicines. It calculated that the "elementary health centres received on the average about 65% of the medicines they should have received", this proportion increasing for the "more developed health centres" and even more for the hospitals. Once in the institutions, "many of the medicines which finally arrive...and which should be distributed freely among patients is taken by health personnel for private use or distribution among friends and relatives. Medicines are also sold to petty traders or directly to patients visiting health workers in their private homes", resulting in a further loss of medicines of 30% for health centres and of 40% for hospitals.

The main consequences of these practices were: underutilization of health services known to be without medicines; a very limited stock of medicines, which forced health professionals to treat patients with inadequate doses of medicines; referral of patients with prescriptions to expensive private pharmacies; and an increase in inequity, as rural populations were clearly at a disadvantage compared to urban populations. The author came to the reluctant conclusion that the root cause of the observed inefficiency was corruption, deeply embedded in socially accepted practices of "gift-giving, with the preponderance of traditional loyalties over obligations to the state, and with a proprietary view of public offices".

The most important single factor encouraging corruption, was, however, "the position of the state as the main source of goods, services and employment and the relative underdevelopment of the private commercial sector". In the end Van Der Geest reluctantly acknowledged that "suggestions to ameliorate the situation are hard to make".

### Reasons for dual practice

With current salary levels in many countries, it is surprising that many people remain in public service, when they could earn much more in private practice. Dual practice allows a standard of living that is closer to what clinical doctors – still a rare resource in many situations – expect, and thus helps retain valuable elements in public service.

Many spend comparatively little time, or none at all, on private practice. It is unlikely that this is only for lack of opportunities, such as a saturated private health care market, or too much competition from the "real" clinicians.

There must be other sources of motivation to keep working in public services. The involvement in (relatively unrewarding) teaching, or in unpaid NGO work shows that other factors – social responsibility, self-realization, professional satisfaction, working conditions and prestige – still play a significant role [3,4,44,45]. A study from Phnom Penh, Cambodia, suggests that the links with the public sector are highly valued, as they give physicians access to information, opinions of influential doctors, recruitment of patients, privileges for treating and referring patients and an opportunity to make a contribution to the community [89].

The gap between income and expectations makes it unavoidable that managers, like other health care workers, will seize opportunities that are rewarding, professionally and financially. Some are of the opinion that dual practice can at times be justified. Health workers in Mozambique and Cape Verde rationalize it: "... to help a sick neighbour" or to help patients "because there are patients that, on account of the long waiting times, do not go to the hospital, they will rather go to these persons in order to avoid wasting their time in the hospitals" [20].

Most, however, implicitly or explicitly condemn such practices while still attempting to explain and/or justify them in various ways. An obvious explanation is that of "serious lack of motivation" and insufficient salaries: "economic reasons, and low salaries ... those are the reasons ... it is a means of surviving" [20].

The reasons for dual practice are not well studied. A number of Portuguese case studies [72,74-76] suggest that these reasons are contextual and vary between professional groups and site of employment (hospital versus health centres). The extent of dual practice seems to vary according to urban or rural residence, according to professional group (it is more common among health workers with university degrees and, for these, more common for doctors than for other professional groups) and within a professional group, according to specialty or occupation. For example, health system managers have fewer opportunities for dual practice than clinicians.

This limited evidence suggests that being a migrant worker, being on temporary contracts and doing shift work are important determinants. This evidence is not conclusive or generalizable, but is welcome as it suggests that dual practice depends not so much on the personal (age and sex), social (marital status) and professional characteristics of health workers – although these are not insignificant – but on factors that are manageable.

Sometimes dual practice may be the unexpected result of health care reform. In Canada, within the public system, designating sites for different levels of surgical acuity during the early stages of regionalization has resulted in a 3.5-fold increase in the number of surgeons working in more than one setting after this restructuring compared to before, as most surgeons do both high- and low-acuity surgeries. This resulted in interference with continuity of care, increased commuting time for both surgeons and medical residents and increased reliance on house staff (with whom surgeons spent less time and are thus less familiar with the limits of their skills) [114].

Another area where provider strain was reported as high was among paid home care staff: low wages, irregular hours, inadequate training and high turnover resulted in lack of continuity of care, staff shortages, waiting lists, health risks to both workers and recipients and impoverishment. Some home workers reported working several jobs to make ends meet [114].

Reforms frequently result in the increase of staff employed on fixed term and temporary contracts [71]. This trend seems to induce dual employment [72,74,75].

### Interventions to deal with dual practice

At the core of the reliance on individual coping strategies, including dual practice, is a very strong motor: the gap between the professional's financial (but also social and professional) expectations and what public service can offer.

Most public responses to individual coping strategies, including dual practice, fail to acknowledge the obvious: that individual employees are reacting individually to the failures of the organizations in which they work, and that these de facto choices and decisions become part of what the organization is.

Adequate responses also imply that the main underlying reason for the observed dual practice can be identified. They call for an understanding of how endemic are the practices observed: Are they isolated, individual cases? Are they specific to the health sector? Or are they widespread in other sectors of society? It is equally important to identify the impact of these practices, particularly the impact in terms of reduced access, inequity and other dangers for the health of the public.

Dual practices have, in some countries, become so prevalent that it has been widely assumed that the very notion of a civil service ethos has completely – and possibly irreversibly – disappeared. But some of the literature reviewed reflects, from the health workers themselves, a conflict between what it means to be an honest civil servant who wants to do a decent job, and the brute facts of life that make them betray that image. The manifest unease that this provokes is an important observation as such. It suggests that even in the difficult circumstances observed in many countries, behaviours that depart from traditional civil servant duty and ethics have not been interiorized as a norm. This ambiguity suggests that interventions to mitigate the erosion of proper conduct would be welcome.

The most relevant conclusion is that there is no single recipe to address the reality of dual practice. Its cause and logic vary, and the resulting differences among situations need to be taken into account in the design of corrective measures [115].

#### What does not work

Pretending that the problem does not exist or that it is a question merely of individual ethics, or approaching it as a problem merely of corruption, does not do justice to the complex nature of the problem and will not make it go away.

Prohibition is equally unlikely to meet with success, certainly if the salary scales remain blatantly insufficient. In situations where it is difficult to keep staff performing adequately for want of decent salaries and working conditions, those who are supposed to enforce such a prohibition are usually in the same situation as those who have to be disciplined. As an isolated measure, restrictive legislation, when not blatantly ignored, only drives dual practice underground and makes it difficult to avoid or correct negative effects [6]. Despite this, governments still resort to prohibition as the main means of controlling dual practice [116].

Closing the salary gap by raising public sector salaries to "fair" levels may not be enough to break the vicious circle. This was attempted by Louro, in Greece, in his restructuring of the health sector in 1945. When public-sector doctors were prohibited by law from pursuing private practice, their average remuneration was raised to take account of their lost income. But there was great resistance by doctors not prepared to give up private practice and professional autonomy [57]. The 1983 Greek NHS Act again required doctors to have a heavily disputed exclusive full-time status in their public sector employment; correspondingly the hospital doctors' salaries were raised so that seniority was favoured – at an average of 112%, or at a range of 11% for junior doctors to almost 211% for directors. Categories such as university and military doctors escaped from the restrictions introduced.

The 1990–1994 reform again allowed doctors to become part-timers if they wished to practice simultaneously in private surgeries or to treat on a per-case basis, but in this case their salaries would be reduced. Notably, though the Ministry of Health expected that around 2500 doctors would become part-timers, only 150 opted for it.

In 1994 an international committee chaired by Abel-Smith reopened this issue, recommending that either rights to private practice would be denied to all doctors working for the NHS or they would be confined to senior doctors for a limited number of sessions. Higher salaries were to be paid to professors to compensate them for their loss of unlimited private practice rights.

In 1997 this still had not been implemented [57]. And it is not a realistic option in many poor countries. In the average low-income country, salaries would have to be multiplied by at least a factor of five to bring them to the level of the income from a small private practice [44,45]. Doing this for all civil servants is unimaginable; doing it only for selected groups would be politically difficult.

Downsizing central bureaucracies and delinking health service delivery from civil service [117] would make it possible to divide the salary mass among a smaller workforce, leaving a better individual income for those who remain. However, experience shows that such initiatives often generate so much resistance among civil servants that they never reach implementation [118]. Where retrenchment becomes a reality it is rarely followed by substantial salary increases, so that the problem remains and the public sector is even less capable of assuming its mission.

Lastly, a mere increase in salary would not automatically reinstate the sense of purpose that is required to make public services function: as such it would not be enough to make moonlighting disappear spontaneously [119,120]. That does not mean that nothing can be done. Improvement is likely to come from a combination of small piecemeal measures that rebuild a proper working environment.

#### Addressing the problem of dual practice openly

A prerequisite is to address the problem of dual practice openly. Where it is not realistic to expect health care workers to dedicate 100% of their time to their public service job, this should be acknowledged. That is the only way to create the possibility of containing and discouraging income-generating activities that present conflicts of interest, in favour of safety valves with less potential for negative impact on the functioning of the health services.

Besides minimizing conflicts of interest, open discussion can diminish the feeling of unfairness among colleagues. It then becomes possible to organize things in a more transparent and predictable way.

There are indications that the newer generations of professionals have more modest expectations and are realistic enough to see that the market for dual practice is finite and to a large extent occupied by their elders. This gives scope for the introduction of systems of incentives that are coherent with the organization's social goals [121].

#### Incentives

Where, for example, financial compensation for work in deprived areas is introduced in a context that provides a clear sense of purpose and the necessary recognition, this may help to reinstate lost civil service values [122]. The same goes for the introduction of performance-linked financial incentives [121]. These can, in principle, address the problem of competition for working time, one of the major drawbacks of dual practice. However, such approaches require well-functioning and transparent bureaucracies, making the countries most in need also those where they are a priori most difficult to implement on a large scale [117,123].

#### Improving working conditions

It makes no sense to expect health workers to perform well in circumstances where the equipment and resources are patently deficient. But improving working conditions involves more than providing an adequate salary and the right equipment. It also means developing career prospects and providing perspectives for training [119,120]. Perhaps most important, it requires a social environment that reinforces professional behaviour free from the favouritism and arbitrariness prevalent in the public sector of many countries.

#### Professional value systems

However ill-defined they may be, the value systems of the professionals are a major determinant in making the difference between good service to the public and bad. It would be naïve to think this could be achieved through mere bureaucratic regulation by governments or donor agencies. With the building up of pressure from donors and from peers as well as from users, civil servant health professionals will be more likely to invest in patterns of behaviours and practices that visibly uphold the professional value system [119,120].

##### Peer pressure

The social and professional culture within a profession may have a major impact on the practice [64,120,121]. Peer influence, building on the concept of group responsibility for self-education and monitoring, as well as multi-component interventions, have been shown to be effective in improving professional practice in the public sector of high-income countries [124,125].

The effect of peer pressure may be positive or negative. Pressure from local practice styles is particularly relevant in situations where there is the most uncertainty concerning the most appropriate treatment protocol [85]. This reflects the practice-styles hypothesis of Wenberg and colleagues in 1982 [126].

Practice styles can be changed through "peer influence meetings", particularly if the change is seen as building up public reputation and status, once more showing that simple income topping-up is not the principal driving force of professional behaviour [127]. This points to the importance, in the absence of effective regulatory mechanisms, of the role of professional societies in ensuring peer-pressure mechanisms to reduce undesirable coping strategies associated with dual practice.

A further possibility is workers' forming peer pressure groups to reduce undesirable coping strategies associated with dual practice. These groups could function to support members to maintain their personal stance as well as to inform the public of their rights. Making public the membership of such a group could be a way of identifying the non-members, an indirect way of increasing pressure [23].

A significant problem with individual coping strategies associated with dual practice is the difficulty of assigning individual responsibilities in situations where these are endemic. In these circumstances it might be relevant to introduce legislation that makes the head of an organization or department legally responsible for the actions of that body. This would be a further means of increasing peer pressure and accountability [23].

##### Pressure from users

Civil society has a particularly important role, specifically in linking reform measures to the experiences and expectations of real people. But civil society must not be seen as a neutral body, particularly in developing countries where patron–client networks or kinship networks have a strong influence on the state and on the patterns of corruption and/or of coping strategies observed. In these situations the reform of civil society itself should be an objective of the interventions to correct such strategies.

In many countries, users/clients/patients are not protected against the consequences of the asymmetry of information they face – with health and financial consequences. From the history of the workers' movement in Europe and as the recent evolution in a number of middle-income countries – such as Thailand's National Forum on Health Care Reform [128] – points out, perhaps the most effective way to help the State regulate professional practice is to increase pressure from civil society. (Fear of malpractice may have a paradoxical effect in that may result in excessive and inappropriate recourse to caesarean sections, for example [85,129].)

Creating opportunities for users to voice their discontent effectively implies that patient's rights must be clear, channels for complaints must be simple, regulatory agencies must be strong and trusted by the public, processes must be explicit and transparent and the judiciary system must be strengthened [23].

#### Recruitment practices

International development agencies, even when they do not have formal, explicit policies regarding dual practice, have become more sensitized to the problem over recent years. This has resulted in a number of recommendations to help minimize the problem. To limit the brain drain due to their own employment policies, organizations such as the World Bank, Norwegian Agency for Development Cooperation (NORAD), German Technical Cooperation (GTZ) or the World Health Organization in principle implement human resources recruitment policies that emphasize the employment of task-specific and short-term consultants, with a commitment of national institutions to retain such staff [90-92,130].

#### Regulating the private sector

The anti-corruption literature, without the necessary empirical evidence to support such claims, actually blames government monopoly of service provision as one of the key determinants of the emergence of some of the coping strategies reviewed above [105]. It has also been argued that the presence of a significant quasi-private system operating within the public sector, i.e. the form of dual practice most common in transitional economies and in developing countries, is detrimental to the development of a strong private sector [23].

The claims for a greater role for the private sector in the provision of health care are based on a number of assumptions that are not all based on empirical evidence and ignore that private practice, in most developing countries, is notoriously unregulated. The fragmentary evidence shows that blanket recommendations regarding the role of the private sector are inappropriate [131].

There is a case for public sector support of the private sector where this serves the public's interest and allows redirection of scarce resources. If that is not the case, support has no rationale. Support, but also mere control, carries costs for the public sector administrative machinery. The costs of the "new" state responsibilities must be compensated for by savings resulting from gains in efficiency and from complementarity [124,131,132].

A key policy question is whether doctors should be allowed to work in both the public and private sectors. As discussed before, prohibition is unlikely to be effective. The real issue is what types of private practice should be allowed in order to minimize conflicts of interest, and what forms of regulatory mechanisms can be introduced to isolate coping strategies that are associated mostly with lack of regulation rather than just with low income [23]. It seems that efforts should be undertaken to ensure multiple and independent channels of accountability, by means of penalties for not satisfying contractual obligations, through channels of accountability to professional councils and associations and to the public.

Regulation is one important factor influencing the coping strategies that result from the interface with the private sector [62,124]. Even when regulations exist, effective enforcement mechanisms are often absent in low- and middle-income countries [125,133]. Therefore, good legislation is not enough. The state must have the means to enforce it. In India, for example, private clinics and mobile teams promote prenatal sex determination by advertising in local newspapers, in spite of government prohibition of the practice [134].

#### Pressure on donors

International collaboration is seen as particularly important regarding the support of international development agencies for actions such as: good-governance interventions in specific domains; supporting methods to curb corruption, including policy dialogue, capacity building, documentation and analysis of best practices and support to national programmes; and making reformers aware of the importance of country conditions in programme development [115].

Anti-corruption strategies have also been approached by donors with different objectives: to reduce poverty, to improve the functioning of democratic institutions, to sustain economic development, political stability and social justice. The lesson for the management of coping strategies and dual practice is that international collaboration cannot be neglected, as donors may be important inducers of coping strategies and dual practice as well as essential partners in the search for solutions.

One way to increase donors' and governments' commitment to deal with the causes of individual coping strategies as well as dual practice might be to include a formal "human resources impact assessment" as a condition for the approval of health projects or components of sector-wide approaches. This could force governments and their partners to face the problems caused by dual practice before it becomes part of the public organization's culture. This would not be a guarantee that it would be effectively dealt with, but might limit the damage [135].

## Conclusions

In terms of sector location, dual practice may be public-on-public, public-on-private or private-on-private. Dual practice is probably present in all countries regardless of income, even in settings where there are major regulatory restrictions, such as China.

Dual practice may lead to predatory behaviour by health workers. This constitutes, in many cases, a de facto financial barrier to access to health care. It delegitimizes public sector health service delivery and jeopardizes the necessary relation of trust between user and provider. Clinicians in dual practice have to compete for patients with themselves, which is an incentive to lower the quality of the care they provide in the public services.

Dual practice contributes also to brain drain, specifically public-to-private brain drain. If public sector medical staff is moonlighting in private practice, this limits access. Besides competition for time, in many cases, the use of the public sector's means of transportation, office infrastructure and personnel represent additional hidden outflows of resources, often in association with dual practice.

This is not the case for managers: involvement in NGO projects or work for donors can foster better coordination in the provision of services, but may constitute a conflict of interest when NGO or project policies are not necessarily congruent with national health policies or the agenda of the public service.

On the other hand, dual practice allows professionals a standard of living that is closer to what they expect, as well as a standard of practice closer to their own perceptions of good professional practice, resulting in higher professional satisfaction.

There is no evidence that dual practice by public sector health professionals complements public practice or promotes greater equity of health care distribution.

The reasons for dual practice are contextual. The extent of dual practice seems to vary according to urban or rural residence, according to professional group (more common among health workers with university degrees and, for these, more common for doctors than for other professional groups) and even within a professional group, according to specialty or occupation. The limited evidence suggests that being a migrant worker, being on temporary contracts and doing shift work are important determinants. This evidence suggests that dual practice depends not so much on the personal (age and sex), social (marital status) and professional characteristics of health workers, although these are not insignificant, but on factors that are manageable.

Sometimes dual practice may be the unexpected result of health care reform. Reforms frequently result in the increase of staff employed on fixed-term and temporary contracts. This trend seems to encourage dual employment.

Therefore, at the core of the reliance on dual practice is the gap between the professional's expectations and what public service can offer. Adequate responses imply the identification of the main underlying reason for the observed dual practice. The most relevant conclusion is that there is no single recipe to address the reality of dual practice.

## Competing interests

The author(s) declare that they have no competing interests.

## Authors' contributions

AB, IF, FH were responsible for the Portuguese case studies. PF and WVLconceived of the study and participated in its design and coordination. All authors read and approved the final manuscript.
